# Adverse Effects of Co-Exposure to Cd and Microplastic in *Tigriopus japonicus*

**DOI:** 10.3390/ijerph192013215

**Published:** 2022-10-14

**Authors:** Wenzhuo Shi, Hao Guo, Junqiang Wang, Xuemeng Han, Wenqian Cai

**Affiliations:** 1 Technical Center for Soil, Agriculture and Rural Ecology and Environment, Ministry of Ecology and Environment, Beijing 100012, China; 2 School of Envirment, Beijing Normal Univeristy, Beijing 100875, China

**Keywords:** microplastic, copepod, co-exposure, reproductive toxicity, reactive oxygen species

## Abstract

There is increasing concern about the adverse impact of exposure to microplastic, as an emerging pollutant, on wild organisms, and particularly on organisms co-exposed to microplastic and other environmental contaminants. It has been widely reported that the combination of microplastics and heavy metals showed obvious toxicity to organisms in terms their growth and development. The present study was performed to determine the impact of binary metal mixtures of cadmium (Cd) and polystyrene microplastic (PS-microplastic) on *Tigriopus japonicus*, a typical marine model organism, using a titration design. Increasing concentrations of PS-microplastic (2 μg/L, 20 μg/L, and 200 μg/L) were titrated against a constant concentration of Cd (15.2 μg/L). The results showed no significant impact of exposure to this dose of Cd or co-exposure to Cd and the lowest dose of PS-microplastic examined (2 μg/L). However, the feeding rate, filtration rate, oxygen consumption rate, and hatching number declined significantly in T. japonicus co-exposed to Cd and higher concentrations of PS-microplastic (20 μg/L and 200 μg/L) (*p* < 0.05). Furthermore, the development of F1 larvae from nauplius stage (N) to adult stage (A) was markedly delayed when co-exposed to Cd and higher doses of PS-microplastic (20 and 200 μg/L), and the effects persisted to the F2 larval stage. Interestingly, the present titration design did not affect the sex ratio or number of oocysts in either the F1 or F2 generation. These results indicated that the current marine environmental concentrations of Cd and microplastic are safe for wild organisms. Further studies are required to address the knowledge gap regarding toxicological effects at the cellular and molecular levels.

## 1. Introduction

The marked increases in plastic production and consumption in recent years have resulted in a serious global plastic pollution problem [1]. As a result of the ongoing breakdown of plastic waste in the environment, the amounts of plastic particles and fragments in the environment have increased exponentially from inland rivers to polar regions [2]. Microplastic, defined as plastic particles < 5 mm in size, is attracting increasing attention due to its wide distribution [1], and there have been many studies of the environmental impacts of microplastic as an emerging contaminant [3,4], especially with regard to its adverse effects on wild organisms and potential ecological damage [5,6].

Previous studies reported that microplastic had adverse effects on the growth, development, and reproduction of organisms [7,8], especially aquatic organisms, as they are similar in size to their natural food and can be taken up easily [3]. For example, Liu et al. [9] reported that *Daphnia pulex* tended to be immobilized after the intake of polystyrene (PS) particles. Moreover, microplastic showed multiple toxic effects in aquatic organisms, such as alimentary canal abrasion or clogging, reduced fecundity, metabolic abnormalities, and death [10,11]. Microplastic was reported to cause a significant decline in the number of offspring and population growth rate of *Daphnia magna* [12]. Similarly, PS-microplastic exposure impeded the growth of goldfish larvae and decreased their swimming speed [13]. Furthermore, PS nanoparticles adhering to the surface of zebrafish embryos could enter the larvae after hatching and spread throughout the whole body through circulation, including the heart and brain [14]. In addition, African catfish exposed to polyvinyl chloride (PVC) microparticles showed less acetylcholinesterase activity in the brain and gill, suggesting neurotoxicity [15]. In addition, the expression of specific genes related to apoptosis and inflammation, including *Casp-3*, *TNF-α*, *IL-1β*, and *IL-6*, increased markedly in mice exposed to PS-microplastic [16]. Changes in the gut microbiota were also commonly observed because of the additives and contaminants carried by microplastic [11]. For example, the diversity and abundance of the intestinal microbiota of the marine medaka (*Oryzias melastigma*) decreased in the presence of PS-microplastic, especially in male fish [17]. Consistent with these findings, the abundance of potentially pathogenic bacteria, *Parabacteroides* and *Alistipes*, was increased in juvenile yellow croaker exposed to nano-PS particles [18].

There have been several recent studies on the toxicity of microplastic combined with other pollutants due to their coexistence in the environment [17,19]. For example, Zhang et al. [20] reported significant decreases in the levels of glutathione peroxidase (GPX) and malondialdehyde (MDA) in *D. magna* co-exposed to PS-microplastic and roxithromycin in comparison to roxithromycin alone. Moreover, PS-microplastic accelerated antibiotic accumulation in *Mytilus coruscus* and synergistically modulated immunotoxic effects [21]. These observations may be explained by the high desorption rate of pollutants adsorbed to microplastic in the alimentary canal of the organisms [22,23]. A positive correlation was reported between the contaminant load in the body and the amount of microplastic ingested by organisms [24,25]. Moreover, laboratory studies showed that the combination of microplastics with heavy metals has greater adverse effects on the growth and development of aquatic and terrestrial organisms than either pollutant alone [17,19].

*Tigriopus japonicus* is a harpacticoid copepod with a wide distribution along coasts worldwide. As a primary predator, it plays a pivotal role in transporting nutrients throughout the marine food chain. Moreover, *T. japonicus* is emerging as a promising model to evaluate the impact of aquatic pollutants in marine ecosystems due to its well-characterized life history, short generation time, predictable population dynamics, and ease of culture in the laboratory. However, there is a paucity of data regarding the adverse impact on *T. japonicus* regarding exposure to microplastic and other contaminations frequently detected in coastal environments, especially heavy metals. We hypothesized that environmentally relevant doses of microplastic and Cd could cause more severe and extensive damage to *T. japonicus* than Cd alone, and transgenerational toxicity was also postulated to occur following co-exposure to these common pollutants. The present study was performed to examine the potential adverse effects of exposure of *T. japonicus* to microplastic and heavy metals using a titration design with feeding, reproduction, and the development of offspring as indicators.

## 2. Materials and Methods

### 2.1. Reagents

Virgin spherical PS-microplastic (10 μm diameter, 10 mg/mL) labeled with fluorescent dyes (excitation wavelength 418 nm; emission wavelength 518 nm) was purchased from BaseLine ChromTech Research Centre (Tianjin, China). Analytical-grade cadmium chloride (CdCl_2_, 99.9% purity) was purchased from Sinopharm Chemical Reagent (Shanghai, China). Stock solutions of PS-microplastic and CdCl_2_ (10 μg/mL) were prepared in ultrapure water.

### 2.2. Copepod Culture

*Tigriopus japonicus* was cultured in an illuminated incubator in our laboratory under the following conditions: water temperature, 20 °C ± 0.5 °C; light intensity, 2100 lux; 12 h:12 h photoperiod. They were fed daily with fresh algae (*I.* *galbana*, 1 × 10^5^ cells/mL), and the culture water was replaced completely every 2 days.

### 2.3. Test Regime in the Laboratory

Preliminary experiments in our laboratory showed that the 48-h LC_50_ of Cd on *T. japonicus* is 15.2 mg/L. Consequently, the most effective dose of Cd was set to 15.2 μg/L to assess the joint toxicity of Cd and microplastic on *T. japonicus* while minimizing the adverse effects of Cd. The groups included a control group (cultured in seawater only), positive control group (15.2 μg/L Cd), and combined groups (15.2 μg/L Cd + 2 μg/L, 20 μg/L, and 200 μg/L PS-microplastic, respectively). The ingestion trial was performed using only 200 μg/L PS-microplastic and Cd + 200 μg/L PS-microplastic. Five replicates were performed for each group, except in the ingestion trial.

#### 2.3.1. Ingestion Test

Exposure solutions were prepared with 1 × 10^5^ cells/mL of *I. galbana* and 200 μg/L PS-microplastic or Cd + 200 μg/L PS-microplastic. These solutions were aliquoted into eight wells of cell culture plates (10 mL/well). Starved *T. japonicus* (F0 generation) were added into the wells (15/well) and each treatment group consisted of eight wells. The plates were cultured in an illuminated incubator (temperature 20 °C ± 0.5 °C; light intensity 2100 lux; photoperiod 12 h:12 h). After 0.5 h, 1 h, 2 h, 4 h, 6 h, 12 h, 24 h, and 48 h, all *T. japonicus* in one well (15/group) were fixed in 4% formaldehyde. After 24 h, the guts of *T. japonicus* were photographed by fluorescence microscopy to observe the ingested microplastic.

#### 2.3.2. Feeding and Oxygen Consumption Test

Exposure solutions were prepared containing *I. galbana* (1 × 10^5^ cells/mL) with the same contaminants, at the concentrations described above, and then poured into plastic bottles (50 mL). Starved *T. japonicus* (F0 generation) were picked out randomly and added to each bottle (30/bottle). The bottles were then sealed to exclude additional air and cultured in the dark for 24 h. The dissolved oxygen (DO) concentration in each bottle was measured with a portable water analyzer. The exposure solution was then fixed with Lugol’s iodine solution. The algae were counted using a hemocytometer under a microscope. The filtration rate and feeding rate, represented by *F* (mL/ind·h) and *G* (cells/ind·h), respectively, were calculated as follows:

(1) $$F=\frac{V}{N}\times \frac{lnC_{t}-lnC_{\mathrm{tf}}}{t}$$

(2) $$G=F\times \frac{C_{\mathrm{tf}}-C_{0}}{lnC_{\mathrm{tf}}-lnC_{0}}$$

where *V* is the volume of solution (mL), *N* is the number of copepods (ind), *t* is the feeding time (h), *C*_0_ is the initial concentration of *I. galbana* (cells/mL), *C*_t_ is the final concentration of *I. galbana* in the control group (cells/mL), and *C*_tf_ is the final concentration of *I. galbana* in the experimental group (cells/mL).

The oxygen consumption rate was also calculated as follows:

(3) $$R_{0}=\frac{\left(C_{0}-C_{i}\right)\times V\times 0.7}{n\times t}$$

where *V* is the volume of solution (mL), *n* is the number of copepods in each bottle (ind), *t* is the experimental time (h), *C*_0_ is the concentration of DO in the control group (mg/mL), *C*_i_ is the concentration of DO in the experimental group (mg/mL), and 0.7 is the coefficient for conversion of oxygen mass to volume.

#### 2.3.3. Growth and Reproduction Test

Exposure solutions containing *I. galbana* (1 × 10^5^ cells/mL) and the contaminants at the concentrations described above were prepared and poured into cell culture plates at 10 mL/well. Newly hatched nauplii (F1 generation) were separated into wells (10 per well) and cultured in an illuminated climate-controlled incubator for 24 d. During the experiment, the exposure solution was replaced with a fresh solution every other day. The growth of *T. japonicus* was observed under a microscope, and the duration of each developmental stage was recorded. The development time was calculated based on the 50% principle. For example, the times when 50% of *T. japonicus* had grown into copepodite stage I and the adult stage were recorded as the start and end of the copepodite stage, respectively. The survival rate and sex ratio of *T. japonicus* were counted once they had grown to the adult stage. Sex was determined by inspecting the organs under a microscope. Ovigerous females were picked out individually and transferred to culture plates (one animal per well). The culture solution was prepared as described for the exposure system. Newly hatched nauplii (F2 generation) were transferred into new wells immediately after observation every day. Finally, oocysts and total nauplii were counted during the 24-d incubation period.

#### 2.3.4. Offspring Development

Nauplii, obtained as described in Section 2.3.3 and cultured in wells (1/well) with 10 other randomly selected individuals in each group, were placed in an illuminated climate-controlled incubator with a culture solution of *I. galbana* (1 × 10^5^ cells/mL) for the evaluation of their development. The growth of *T. japonicus* was observed under a microscope every day, and the culture solution was changed every other day until nauplii had grown into the adult stage. The developmental time of each stage was also recorded according to the 50% principle as described above. Similarly, the survival rate and sex ratio were calculated for each well once the adult stage had been reached.

#### 2.3.5. Nutritional Analysis

The newly hatched nauplii were transferred into beakers (100/beaker) and cultured in an illuminated climate-controlled incubator with an exposure solution of *I. galbana* (1 × 10^5^ cells/mL) and the contaminants, at the concentrations described above. The 70% exposure solution was changed every other day until the nauplii reached the adult stage. Then, adult *T. japonicus* were transferred into Eppendorf tubes with 50 μL of 0.9% physiological saline. The cells were disrupted with ultrasound for 5 min and centrifuged (5000 rpm/min, 4 °C, 10 min). The protein content of the supernatant was measured using Coomassie brilliant blue G-250 (Bradford, 1976), and triglyceride (TG) and glucose (GLU) were measured using assay kits.

### 2.4. Statistical Analysis

Image J (NIH, Bethesda, MD, USA) was used to process the photographs and calculate fluorescence intensity. Data are expressed as the mean ± standard error. Statistical analyses and data plotting were performed using SPSS Statistics 25 (SPSS Inc., Chicago, IL, USA) and SigmaPlot 12.5 (Systat Software, Richmond, CA, USA). The one-way analysis of variance (ANOVA) was performed to compare the treatments, and *p* < 0.05 was taken to indicate statistical significance.

## 3. Results

No significant mortality of *T. japonicus* was observed with exposure to Cd alone or Cd plus a low concentration of PS-microplastic. However, a few test organisms died when co-exposed to Cd and a high concentration of PS-microplastic (Table 1). Thus, no acute toxicity occurred in these exposure trials.

### 3.1. Impact on Physical Activities

**Figure 1:**
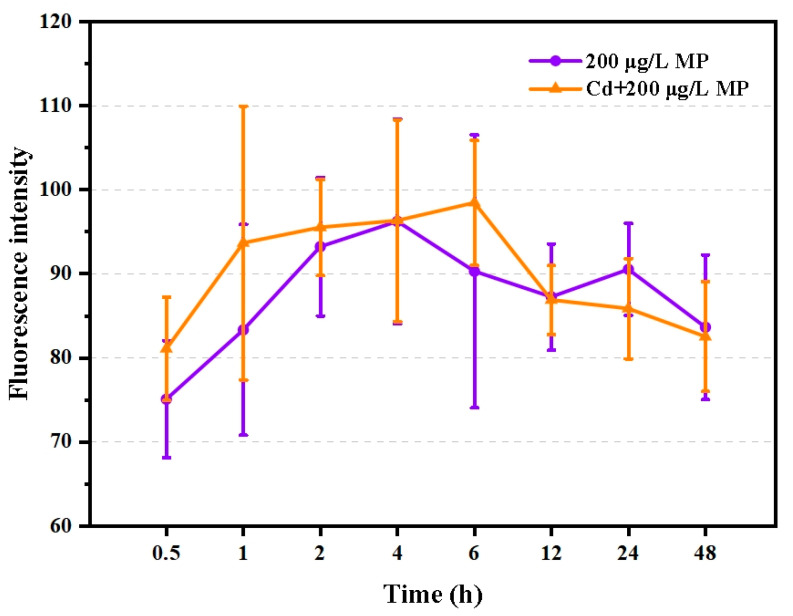
Fluorescence intensity of *T. japonicas* (F0 generation) exposure to single PS-microplastic and co-exposure with Cd (*n* = 6).

No significant differences in microplastic ingestion were found between *T. japonicus* co-exposed to 15.2 μg/L Cd and 200 μg/L PS-microplastic and those exposed to 200 μg/L PS-microplastic alone (*p* > 0.05), suggesting that this dose of Cd did not hamper the ingestion of *T. japonicus*. Interestingly, the amounts of PS-microplastic ingested, as indicated by the fluorescence intensity, increased rapidly until 6 h (Figure 1), indicating that PS-microplastic of the size used in the present study was easily ingested by *T. japonicus*, especially the following starvation. However, the amount ingested declined from 6 h to 48 h (Figure 1). This may be explained by satiety and the balance between ingestion and excretion.

**Figure 2:**
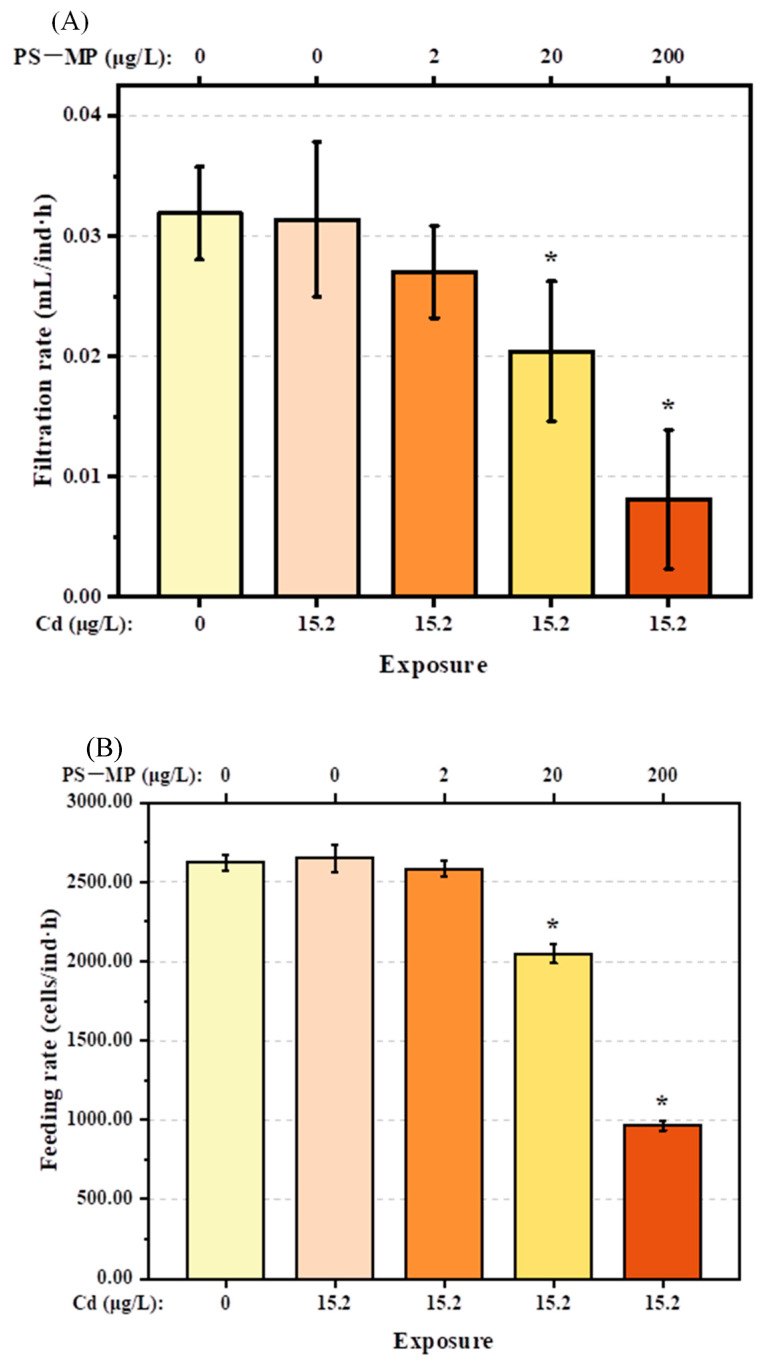

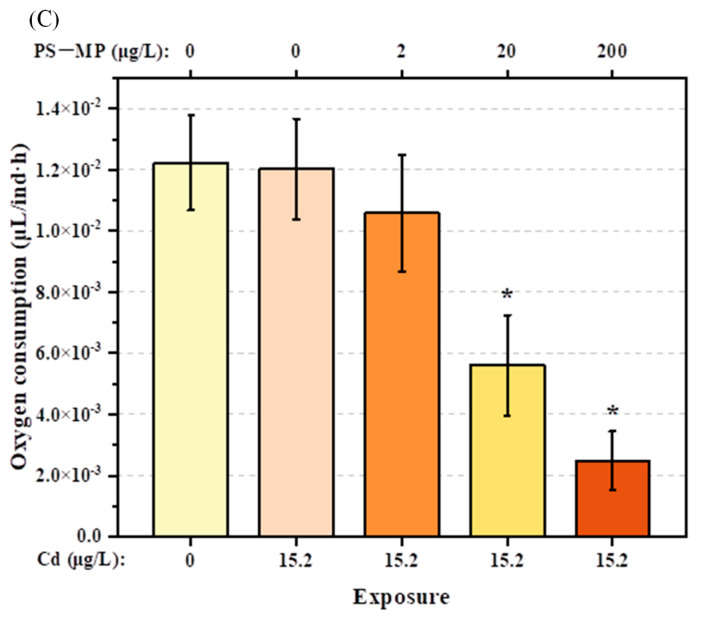
Physical activities of *T. japonicas* (F0 generation) exposure to Cd and PS-microplastic (*n* = 5). (**A**) Filtration rate, (**B**) feeding rate, and (**C**) oxygen consumption. Significant differences between the exposure and control groups are marked with * (*p* < 0.05).

In comparison to the control group, Cd alone at the dose used in the present study (15.2 μg/L) had no significant adverse effects on the filtration rate, feeding rate, or oxygen consumption of *T. japonicus* (*p* > 0.05). However, these behaviors decreased gradually with increasing PS-microplastic concentration, although the effects were not evident in *T. japonicus* co-exposed to Cd and the lowest concentration of PS-microplastic examined in this study (*p* > 0.05) (Figure 2A–C). The oxygen consumption rate decreased rapidly to 45% and 20% in *T. japonicus* exposed to Cd + 20 μg/L PS-microplastic and Cd + 200 μg/L PS-microplastic, respectively (*p* < 0.05); similar results were found for the filtration and feeding rates when the PS-microplastic concentrations increased to 20 and 200 μg/L with a constant concentration of Cd (*p* < 0.05). In addition, significant reductions in the filtration rate (by 75%) and feeding rate (by 63%) were observed in the Cd + 200 μg/L PS-microplastic treatment group.

### 3.2. Impact on the Development of T. japonicas

**Figure 3:**
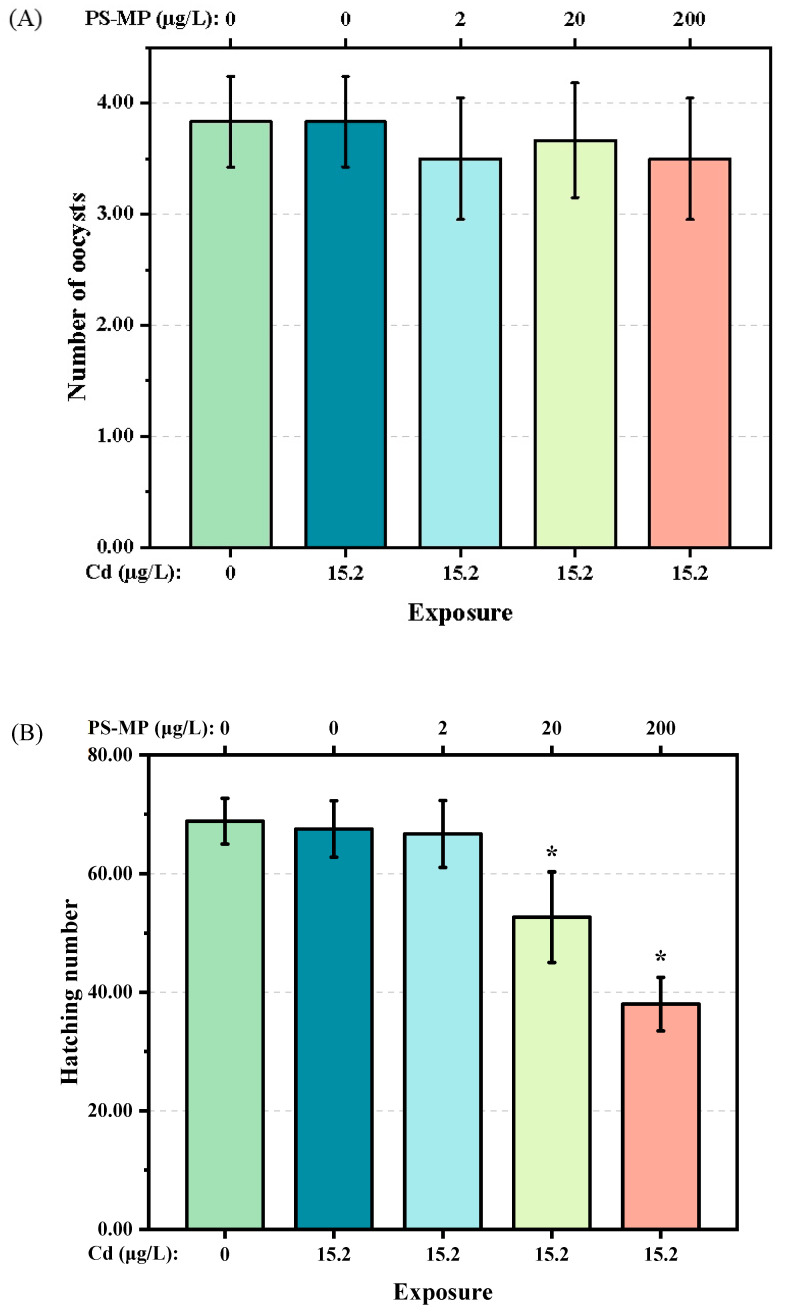
(**A**): Number of oocysts of the F1 generation and (**B**) hatching number of the F2 generation of *T. japonicas* exposure to Cd and PS-microplastic. The asterisks (*) indicate statistical differences compared with control group(*p* < 0.05).

The number of oocysts of F1 generation *T. japonicas* exposed to either single Cd or the mixture of Cd + 2 μg/L PS-microplastic, which ranged from 3 to 4, did not decrease significantly compared with the control group (*p* > 0.05) (Figure 3A). However, slight decreases were found in *T. japonicas* exposed to the mixture of Cd + PS-microplastic at higher concentrations of 20 and 200 μg/L. However, the hatching number of the F2 generation was decreased following exposure to Cd + 20 and 200 μg/L PS-microplastic (*p* < 0.05) (Figure 3B), indicating a decreasing trend according to the dose of PS-microplastic. Notably, the hatching number of the F2 generation following exposure to 200 μg/L PS-microplastic was about 38 ± 4.52, corresponding to 55% of the control group.

**Figure 4:**
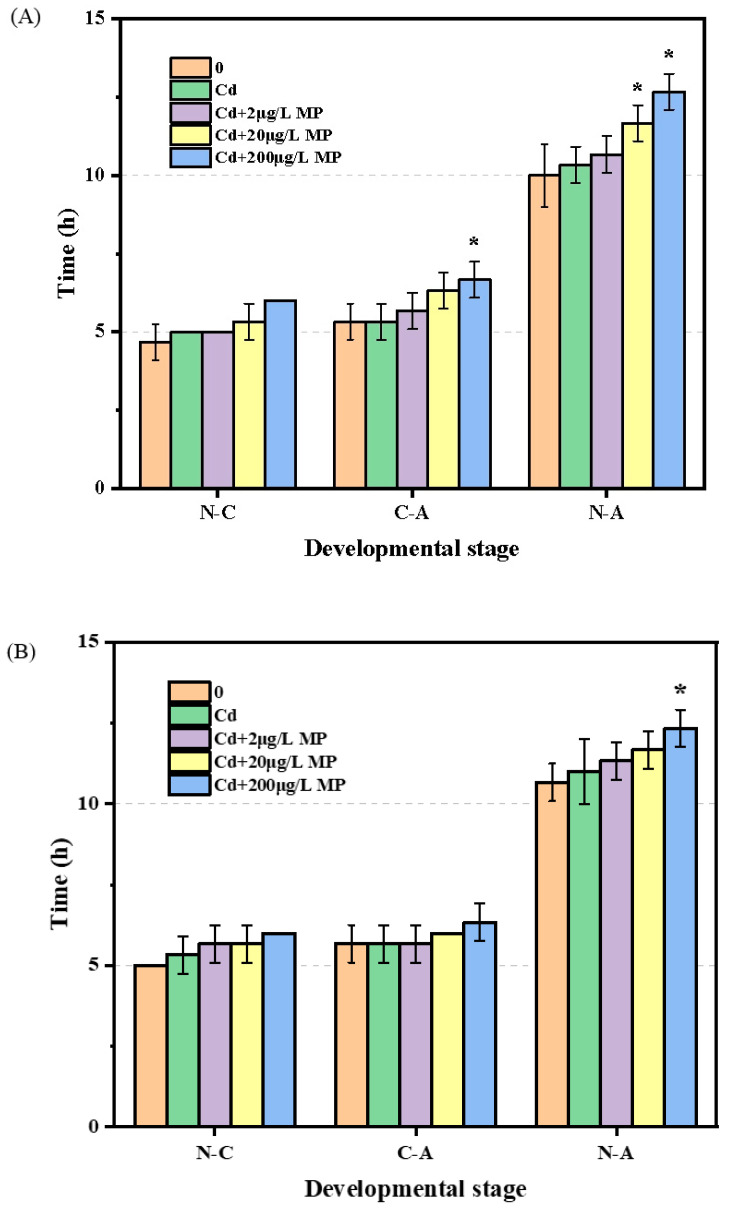
Development of *T. japonicas* (N-C, C-A, and N-A) exposure to Cd and microplastic. (**A**) F1 generation and (**B**) F2 generation. Significant differences between the exposure and controlled groups are marked with * (*p* < 0.05).

Furthermore, neither single Cd exposure at a dose of 15.2 μg/L nor co-exposure to Cd and the lowest PS-microplastic concentration of 2 μg/L affected the development of F1 and F2 *T. japonicas* at any developmental stage (*p* > 0.05) (Figure 4A,B). Moreover, the nauplius to copepodid (N-C) stage in F1 and F2 generations were not impacted by any of the Cd + PS-microplastic doses (*p* > 0.05). Interestingly, however, the growth of the copepodid to adult (C-A) stage was significantly delayed from 5.33 to 6.67 days in the F1 generation following co-exposure to Cd and the highest dose of PS-microplastic examined here (200 μg/L) (*p* < 0.05), equivalent to 125% of the corresponding control, but no such delay was observed in the F2 generation (*p* > 0.05). Overall, the growth time of the nauplius to adult (N-A) stage was lengthened in the F1 generation co-exposed to Cd + 20 and 200 μg/L PS-microplastic by 1.67 and 2.67 days, respectively (*p* < 0.05), but that was only observed in the F2 generation with co-exposure to Cd + 200 μg/L PS-microplastic (1.67 days; *p* < 0.05).

**Figure 5:**
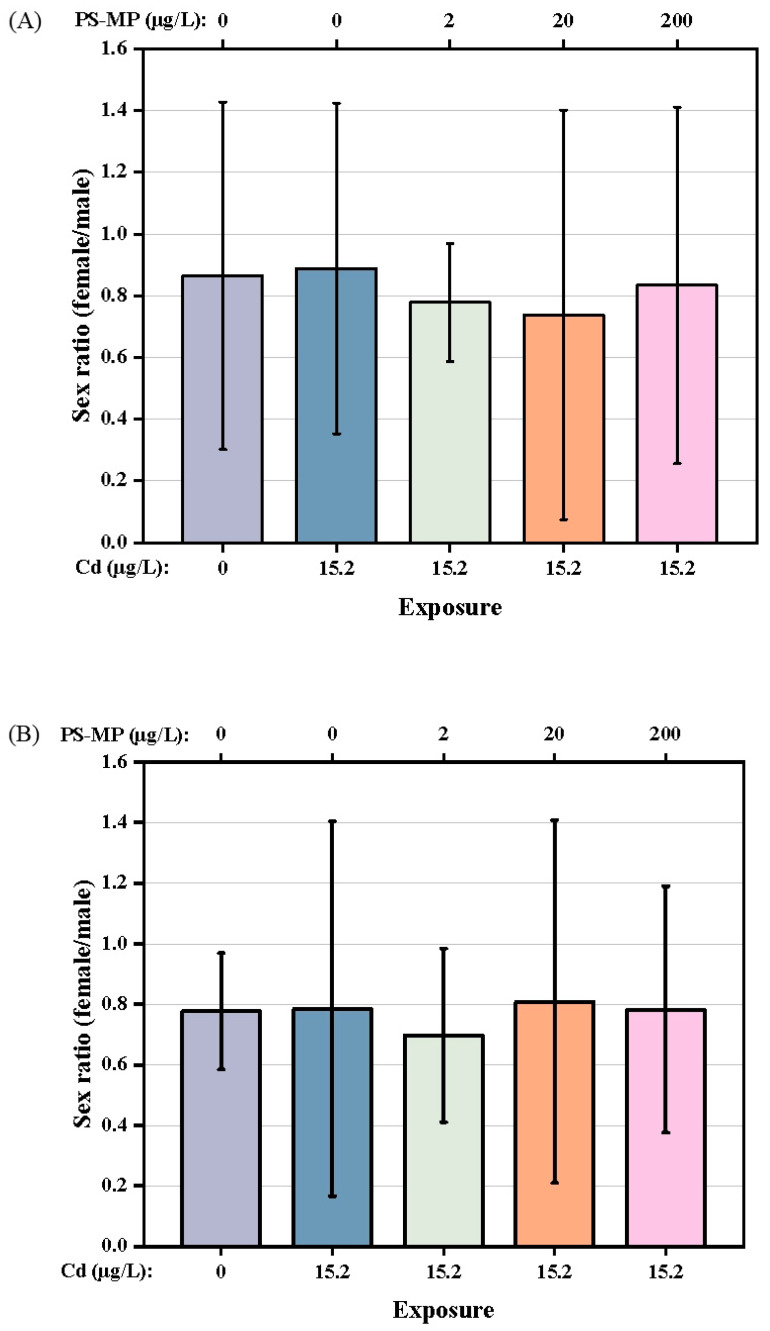
Sex ratio of *T. japonicas* exposure to Cd and PS-microplastic. (**A**): F1 generation, and (**B**): F2 generation.

In addition, the female/male sex ratio of F1 and F2 generation *T. japonicas* (about 0.82 and 0.77, respectively) was not affected by either exposure to Cd alone or co-exposure to Cd + PS-microplastic at any dose examined (*p* > 0.05) (Figure 5A,B). The exposures had no influence on the survival rate of the parental copepods (Table 1). However, the Cd + 200 μg/L PS-microplastic treatment group showed a significant decrease in offspring survival (86.67% compared to the control).

### 3.3. Impact on Nutritional Components

**Figure 6:**
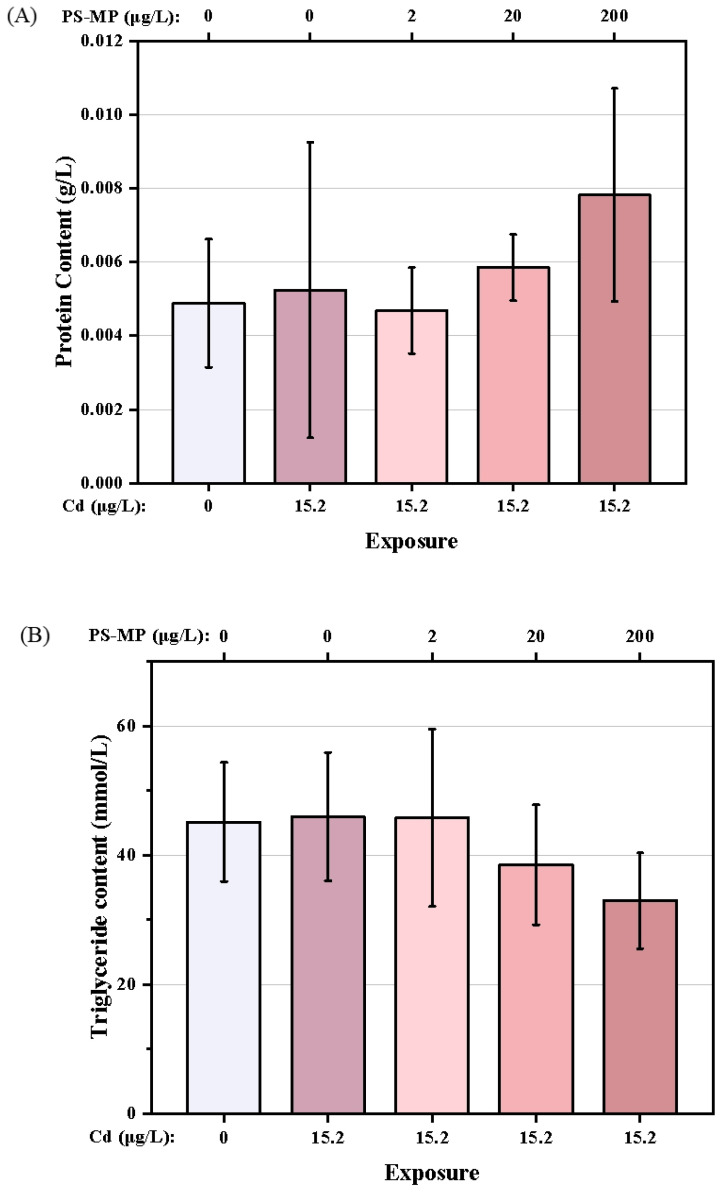

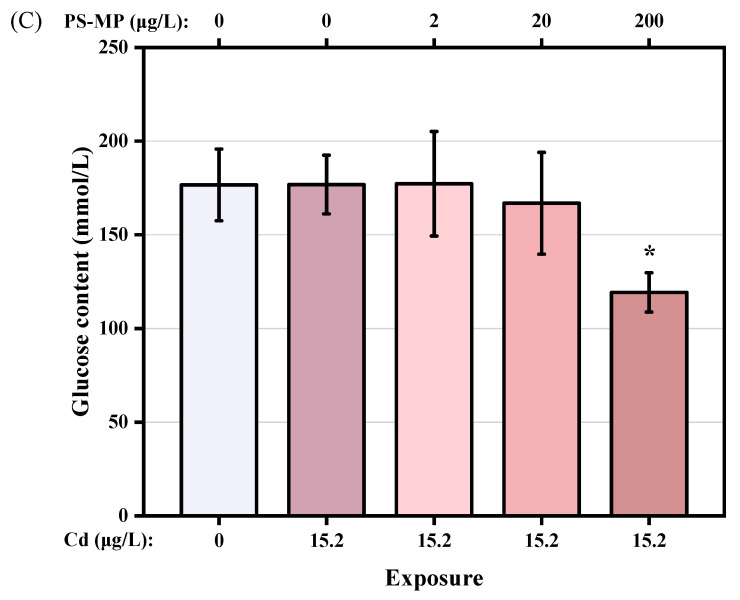
Nutritional components of *T. japonicas* exposure to Cd and microplastic. (**A**) Protein content, (**B**) Triglyceride content, and (**C**) Glucose content. Significant differences between the exposure and controlled groups are marked with * (*p* < 0.05).

The nutritional components of each treatment are shown in Figure 6A–C. Compared with the control group, Cd treatment was associated with a slight increase in the TG content of the copepods. However, the TG content decreased with successive increases in PS-microplastic concentration, with a 27.0% decrease seen in the Cd + 200 μg/L PS-microplastic cotreatment group compared to the corresponding control. The GLU content also showed a dose–effect relationship, with a significant decrease of 32.5% seen in the Cd + 200 μg/L PS-microplastic cotreatment group compared to the corresponding control. (*p* < 0.01). There were no reductions in nutritional components but a slight increase in GLU content on cotreatment with 2 μg/L PS-microplastic compared to exposure to Cd alone, indicating that a low concentration of PS-microplastic did not significantly affect the nutritional components of the copepod.

## 4. Discussion

Microplastics and heavy metals are common pollutants in the marine environment. The toxicity of these pollutants to wild marine organisms is a matter of concern in marine ecotoxicology. PS-microplastic is hard to degrade in the intestines of organisms. While the mechanical abrasion of the intestinal tract may break the microplastics into smaller pieces, bring more potential risk. As a typical environmental pollutant, Cd can rapidly accumulate both directly and indirectly in aquatic organisms through the food chain. Furthermore, environmental microplastic can also transport other pollutants, including Cd, along food webs through intentional and unintentional ingestion [10,23], which may enhance the adverse impacts of these pollutants on animals and humans.

### 4.1. Physical Impact of PS-Microplastic and Cd

While the single dose of Cd (15.2 μg/L) showed no significant effects (*p* > 0.05), cotreatment had adverse effects on *T. japonicus* in a concentration-dependent manner, including decreased filtration and feeding rates, and oxygen consumption, as well as delayed growth and reproductive failure. Similar results were reported previously for polyethylene (PE) and polyamide-nylon 6 (PA 6) microplastics, which also negatively influenced the feeding of *T. japonicas* in a dose-dependent manner [26]. Similarly, a recent study reported that the heart rate of zebrafish embryos was significantly decreased when exposed to PS-microplastic, alone or admixed with Cd, in a PS-microplastic dose-dependent manner [27].

Earlier studies suggested that the adverse impacts of co-exposure to heavy metals and microplastic were mainly due to the accumulation of heavy metals rather than microplastic [17]. In the present study, the concentration of Cd was sufficiently low to avoid any toxic effects from the heavy metals, and all of the indexes examined here were decreased by cotreatment with PS-microplastic. These observations suggested that the interaction between Cd and PS-microplastic exacerbated the toxicity. Wang et al. [10] reported that microplastics could act as vectors for metals. Cd and other metals adsorbed into microplastic are transferred into organisms through ingestion and then desorb in the gastrointestinal environment. More metals are desorbed from large microplastic particles due to their long residence time in the alimentary system [23]. Marine medaka exposed to microplastic and heavy metals showed higher tissue Zn concentrations than those exposed to heavy metals alone [17]. Therefore, microplastic increased Cd intake and amplified its effects on *T. japonicus*, where Cd ions can enter the cells through calcium channels and subsequently cause intracellular toxicity [28]. Zhang et al. [27] reported that the body length of zebrafish larvae also decreased with Cd + PS-microplastic co-exposure, except when Cd was absent, indicating the toxic effect of Cd combined with PS-microplastic on zebrafish larval development. In addition, the Cd + PE-microplastic mixture had greater toxicity in the cladoceran *Moina monogolica* than PE microplastic alone [10]. These observations suggest that the presence of Cd or other heavy metals in the environment enhances the toxicity of microplastic to aquatic organisms. This effect may depend on the types of microplastic and heavy metals, as well as the species of organisms, given the differences in toxicological pathways. Further studies are required to determine the underlying mechanisms of these effects.

### 4.2. Impact on Reproductive Capacity of T. japonicus of Cd and PS-Microplastic

There is accumulating evidence that microplastic can impair the reproductive capacity of organisms, for example by reducing fertilization and inhibiting the development of offspring [26]. PS-microplastic disrupts the hypothalamic–pituitary–gonad (HPG) axis of the marine medaka and affects the hatching rate and development of the offspring [17,29,30]. The potential influence on offspring of prenatal microplastic exposure merits further study, as organisms are widely considered to be more sensitive to toxins in the early developmental stages.

Interestingly, the reproductive outcomes of *T. japonicus* differed among groups, although not significantly. Development was delayed in a PS-microplastic concentration-dependent manner (Figure 4A,B). Compared with the N-C stage, exposure was more toxic to *T. japonicus* in the C-A stage, consistent with the previous finding that pre-adults were more sensitive to several heavy metals than nauplii and copepodites [31]. The offspring of marine organisms showed a delay in development following the exposure of the parental generation to microplastic [26,32]. Similarly, the highest concentration of PS-microplastic in our experiments caused significant developmental delays in both the parental generation and offspring. The results showed that the adverse impact of co-exposure to Cd and microplastic on the development of *T. japonicas* persisted into the F2 generation, although the effect was less severe than in the F1 generation. A previous study showed that exposure to 5 μg/L Cd delayed copepod development [33]. However, in this study, there was no apparent increase in the N-C stage after treatment with 15.2 μg/L Cd (*p* > 0.05). We speculated that PS-microplastic was the main factor contributing to the observed toxic effects. For example, PS-microplastic prolonged the developmental time of *T. japonicus* in a dose-dependent manner.

Microplastic intake alters the nutrient metabolism of aquatic organisms [11]. Exposure to PS-microplastic also affected the energy reserve and nutritional components of the marine jacopever, *Sebastes schlegelii* [6]. Nutritional deficiency can occur due to the decreased lipid accumulation, which in turn was caused by the ingestion of microplastic instead of *I. galbana*; this delayed the development of *T. japonicus* and even impaired fertility.

Many surveys have shown that microplastic reduces the reproductive capacity of aquatic organisms. For example, the presence of microplastic impaired the sperm velocity and gamete fusion efficiency of the broadcast spawning bivalve *Tegillarca granosa* [34], thus reducing the hatching rate. In the present study, there was no change in the number of oocysts in any treatment group compared to the control (Figure 3A). However, the effect of PS-microplastic concentration on hatching number was highly significant (*p* < 0.01), with marked reductions seen in the two groups exposed to higher doses of PS-microplastic (Figure 3B). We speculate that the change in hatching number was due to a decrease in viability of the zygote induced by PS-microplastic. These findings provided evidence for the reproductive toxicity of microplastic, further supporting the results of previous studies reporting large numbers of unfertilized egg sacs in *T. japonicas* exposed to PS-microplastic [5] and the reduced hatching rate of *O. melastigma* following exposure to 20 μg/L PS-microplastic [29]. However, the number of offspring of *D. magna* exposed to 5 mg/L showed no significant difference from the controls [35], indicating that 20 and 200 μg/L PS-microplastic contributed to the marked decrease in hatching number in the present study, through incompletely. In addition, the co-exposure to Cd and microplastic reduced the number of offspring in first broods, the number of broods, and the total reproductive capacity of *M.* *monogolica* [10]. It could be deduced that the increased Cd concentration seen in our study may have enhanced the impact of PS-microplastic on the test organisms.

The exposure conditions in the present study had no effect on the sex ratio of the copepod (*p* > 0.05), although PS-microplastic exposure slightly decreased the female/male ratio overall, as shown in Figure 5. These observations suggest that PS-microplastic may have greater toxicity to females than males in both generations; moreover, the copepod was more sensitive to the effects of PS-microplastic in the presence of Cd. These results corroborated the findings of Park et al. [8], i.e., that the sex ratio of newly birthed mice changed when parental mice were exposed to microplastic. In addition, microplastic had greater effects on sex hormone balance in female than male marine medaka [29]. Previous studies suggested that microplastic can be transferred from parents to offspring. PS beads with a diameter of 0.5 μm had no evident effect on the survival rate of the parental generation at a concentration of 25 μg/mL, but the survival of the offspring was significantly decreased [5]. Moreover, exposure to microplastic reduced the fecundity of *D. magna* and increased the mortality of the offspring, where these toxic effects persisted for more than three generations [12]. In our experiment, Cd + 20 μg/L PS-microplastic co-exposure was associated with a mortality rate of 3.33% for the offspring, with no change seen in the mortality rate for the parental generation (Table 1); this indicates that a high concentration of PS-microplastic is more lethal for offspring. This study supported a previous finding of increased zebrafish mortality with exposure to higher concentrations of microplastic [27].

There is accumulating evidence that microplastic exposure causes various types of reproductive toxicity. Firstly, the quantity and quality of germ cells are decreased, reflected in smaller germ cells and lower sperm speed [7,16]. Fertility was also decreased with delayed hatching time, which reduced the fertilization success rate and the number of eggs produced [9]. Furthermore, adverse effects of microplastic exposure have been observed on embryos. Experiments on zebrafish embryos showed that microplastic can adhere to and even cross the chorion of the embryo, enter the yolk sac, and induce a hypoxic microenvironment in the embryo [14,36]. Nanoplastics also induce a hypoxic microenvironment in zebrafish embryos [14]. These processes may all contribute to the adverse effects on offspring. This study provided preliminary support for the hypothesis that PS-microplastic has transgenerational toxic effects in *T. japonicus*.

Microplastics absorb and carry various contaminants due to their large specific surface area, hydrophobic surface, and the large number of adsorption sites. The sorption equilibrium time between Cd and PS-microplastic is less than 24 h [37]. In this research, the ingestion test showed that the copepod ingested a large amount of PS-microplastic. As a result, Cd was brought into the organisms. Previous studies showed that microplastic promotes the aggregation of Cd in the tissues of aquatic organisms, where the degree of accumulation is dependent on the microplastic concentration [10,19]. This may be a fundamental reason for the adverse effects of microplastic on *T. japonicus*. The evaluation of the ecotoxicity of microplastic requires consideration of the effects of co-exposure with heavy metals. Recent studies showed that microplastic induces reactive oxygen species accumulation in *T. japonicus* and other copepods, thus increasing oxidative stress [16,29]. These observations suggest that exposure to high microplastic concentrations could impair the inflammatory response, leading to developmental and reproductive toxicity. Further studies are required to determine whether Cd and PS-microplastic show synergistic toxic effects, and to elucidate the underlying mechanisms.

## 5. Conclusions

PS-microplastic had various toxic effects on *T. japonicus*, including decreased viability and fertility, and delayed development. Exposure in early life stages resulted in adverse effects on the offspring. Most of the toxic effects of PS-microplastic were dose-dependent. These findings suggest that transgenerational toxicity in this marine copepod is a major problem. Considering the crucial position of *T. japonicus* in the food chain, the potential risk to organisms at higher trophic levels (through biomagnification) is of concern. Further studies are required to determine the combined effects of PS-microplastic and other pollutants, particularly in terms of transgenerational toxicity.

## Figures and Tables

**Table 1:** Survival rate of *T. japonicas* in F0 and F1 generation exposed to Cd and PS-microplastic (*n* = 10).

Treatment	Survival Rate (%)	
	F0	F1
Control	100.00	100.00
Cd (15.2 μg/L)	100.00	100.00
Cd (15.2 μg/L) + 2 μg/L MP	100.00	100.00
Cd (15.2 μg/L) + 20 μg/L MP	100.00	96.67 ± 5.77
Cd (15.2 μg/L) + 200 μg/L MP	93.33 ± 5.77	86.67 ± 5.77
